# Strong paleoclimatic legacies in current plant functional diversity patterns across Europe

**DOI:** 10.1002/ece3.2131

**Published:** 2016-04-18

**Authors:** Alejandro Ordonez, Jens‐Christian Svenning

**Affiliations:** ^1^ Section for Ecoinformatics and BiodiversityDepartment of BioscienceAarhus UniversityNy Munkegade 114DK‐8000Aarhus CDenmark

**Keywords:** Climate velocity, climatic change, ecosystem function, functional dispersion, functional richness, functional traits, glacial–interglacial, historical climate, species richness‐mediated effects

## Abstract

Numerous studies indicate that environmental changes during the late Quaternary have elicited long‐term disequilibria between species diversity and environment. Despite its importance for ecosystem functioning, the importance of historical environmental conditions as determinants of FD (functional diversity) remains largely unstudied. We quantified the geographic distributions of plant FD (richness and dispersion) across Europe using distribution and functional trait information for 2702 plant species. We then compared the importance of historical and contemporary factors to determine the relevance of past conditions as predictors of current plant FD in Europe. For this, we compared the strength of the relationships between FD with temperature and precipitation stability since the LGM (Last Glacial Maximum), accessibility to LGM refugia, and contemporary environmental conditions (climate, productivity, soil, topography, and land use). Functional richness and dispersion exhibited geographic patterns with strong associations to the environmental history of the region. The effect size of accessibility to LGM refugia and climate stability since the LGM was comparable to that of the contemporary predictors. Both functional richness and dispersion increased with temperature stability since the LGM and accessibility to LGM refugia. Functional richness' geographic pattern was primarily associated with accessibility to LGM refugia growing degree‐days, land use heterogeneity, diversity of soil types, and absolute minimum winter temperature. Functional dispersion's geographic pattern was primarily associated with accessibility to LGM refugia growing degree‐days and absolute minimum winter temperature. The high explained variance and model support of historical predictors are consistent with the idea that long‐term variability in environmental conditions supplements contemporary factors in shaping FD patterns at continental scales. Given the importance of FD for ecosystem functioning, future climate change may elicit not just short‐term shifts in ecosystem functioning, but also long‐term functional disequilibria.

## Introduction

Contemporary climatic conditions and environmental heterogeneity have been shown to be important drivers of European floristic diversity (Bakkenes et al. 2002; Thuiller et al. 2005; Svenning et al. 2010). Nonetheless, it has also been demonstrated that climatic changes since the LGM (Last Glacial Maximum, ~21,000 year BP) are significant predictors of European floristic diversity, mainly due to their effects on species ranges via dispersal constraints (Willner et al. 2009; Normand et al. 2011). Moreover, the impact of Neogene climate changes has also been shown to constrain the current European floristic diversity via complete lineage losses (Svenning 2003; Eiserhardt et al. 2015). The prevalence of historical legacies in European plant distributions (Willner et al. 2009; Normand et al. 2011; Dullinger et al. 2012) and richness (Svenning et al. 2010) due to changes in the stability, location, and extent of suitable conditions since the LGM is well documented. The prevalence of such historical legacies implies that the same factors could have also shaped current functional diversity (FD; defined as the range and variability in the trait composition of a local community or region; Díaz et al. 2007) in the region.

The importance of contemporary conditions for FD has been studied at large spatial scales in both the New World (e.g., Swenson and Weiser 2010; Swenson et al. 2012a; Šímová et al. 2014; Ordonez and Svenning 2016) and Europe (e.g., Petchey et al. 2004; Thuiller et al. 2006, 2014; Villéger et al. 2013; Mathieu and Davies 2014; Ordonez and Svenning 2015). By comparison, the degree to which environmental changes over the late Quaternary still shapes FD has only until recently been considered. For example, Ordonez and Svenning (2015) evaluated the prevalence of a lower than expected functional richness and variability of European angiosperms, showing that areas covered by ice at the end of the LGM had larger functional deficits than areas considered as refugia during the same period. However, to the best of our knowledge, the relative importance of historical factors as predictors of the realized European plants FD when compared to contemporary factors remains untested (but for efforts in this direction in the New World, see Ordonez and Svenning 2016). Knowing how contemporary and historical conditions individually and jointly determine FD across continents would help to determine how species, communities, and ecosystems may respond to future climatic conditions (Chapin et al. 2000; Van Bodegom et al. 2012).

There are several mechanisms through which FD patterns can be affected by past environmental changes, most of which have been linked to limitations on effective dispersal, environmental or biotic filtering, and diversification rates. First, the location of glacial refugia has shaped current distribution patterns via dispersal‐limited recolonization dynamics, modulated by trait‐linked differences in dispersal capacity (Willner et al. 2009; Normand et al. 2011; Dullinger et al. 2012) and migration rates (Davis and Shaw 2001). Second, glacial climate may have caused past filtering of the species pool of unsuitable species according to their functional attributes (cf. Svenning 2003), in a way similar to that reported for North America based on contemporary conditions (Swenson et al. 2012a; Šímová et al. 2014). Third, the effect of biotic interactions on range dynamics can impose constraints to range expansion rates and realized species assemblages (Van der Putten 2012; Wisz et al. 2013). Last, glacial climates may have induced trait‐linked changes in diversification rates (Dynesius and Jansson 2000).

Focusing on the currently realized FD of European plants, we answer two questions related to the importance of historical factors as predictors of European FD patterns: (1) Do current geographic patterns in plant FD across Europe exhibit imprints of environmental changes during the late Quaternary?; and (2) To what extend the influence and importance of historical imprints as predictors of FD patterns are comparable to that of contemporary factors?. We hypothesize that high functional richness and low dispersion would be observed in assemblies where historical conditions show high climatic stability (measured as climate velocities; *see Methods*) that are close to areas considered as refugia at the end of the LGM (measured as accessibility; *see Methods*). These conditions would be as important as those contemporary conditions considered to promote high species diversity (e.g., high water–energy availability and/or a large habitat heterogeneity; cf. Weiser et al. 2007 and; Kerkhoff et al. 2014).

Our reasoning is that high environmental stability and proximity to LGM refugia promote a wider trait space sampling due to stability in the species richness and composition over time (Mouillot et al. 2013). These conditions also reduce extinction risk (Dynesius and Jansson 2000), resulting in less pruning of the functional space. Furthermore, environmental stability would also promote the functional differentiation among co‐occurring species via the functional specialization of species over time. As a result, we expect narrower niches, leading to equal trait spacing among species (Scheffer and van Nes 2006) and low dispersion of the species assemblage trait composition (Mouillot et al. 2013).

## Materials and Methods

### Distribution data

Using the AFE (Atlas Florae Europaeae), we characterized the distribution of European angiosperms. We focused on European angiosperms as its current ecology, and historical distribution is relatively well understood and to minimize the effect of different evolutionary trajectories in the region (i.e., the dominance of gymnosperms in higher latitudes). The AFE maps the distribution of European flora on an equal‐area mapping unit of ~50 × 50 km (AFE grid cells; Jalas and Suominen 1994–1999) based on the Universal Transverse Mercator projection and Military Grid Reference System. We excluded observations for the former Soviet Union due to the incomplete recording of this area. Selected AFE grid cells were the geographic units for all the computations and analyses performed in this study.

We used 2702 species (included in 21 orders, 72 families, and 461 genera) listed in AFE as occurring in continental Europe. Forms, varieties, and subspecies were collapsed to species. We are aware of the taxonomic limitations of the AFE (it only covers ~25% of the plant species in Europe) and that it is biased against woody plants. To ensure that our estimates were robust to the used species pool, we evaluated the consistency in the geographic trends in our estimates of FD for seven orders of European angiosperms with a pan‐European distribution (Appendix S1). We observed highly correlated FD patterns across orders, suggesting that analyses based on AFE data can provide a general description of the FD trends in the region.

### Trait information and functional diversity estimation

We used five ecomorphological traits: specific leaf area (cm^2^*g^−1^), seed mass (mg), maximum stem height (m), wood density for woody species or stem‐specific density for nonwoody species (WD; kg*m^−3^), and growth form (ferns, graminoids, forbs, shrubs, trees, climbers). These traits were selected due to their importance for characterizing plant functional strategies (Wright et al. 2004; Chave et al. 2009), the known link between these traits and abiotic environments (Wright et al. 2005; Ordonez et al. 2009; Swenson and Weiser 2010; Swenson et al. 2012a), and the frequent use of these traits to predict the geographic distribution of vegetation types and ecosystem functions (Díaz and Cabido 2001; Lavorel and Garnier 2002; Lavorel 2013). The limitations of using a small set of traits to characterize the effects of present and historical environmental conditions of FD are well known. However, the selected attributes represent a subset of those widely reported in the literature allowing us to have information on a large number of species.

Mean trait values for the study species were initially determined using multiple trait databases (Wright et al. 2004; Moles and Westoby 2006; Kleyer et al. 2008; Liu et al. 2008; Chave et al. 2009; Ordonez et al. 2010; Kattge et al. 2011; Ordonez and Olff 2013). Gaps in the database (proportion of missing values for specific leaf area: 50%, maximum stem height: 53%, seed mass = 50%: stem/wood density: 42%) were filled using an evolutionary (based on genus) and ecological (based on growth form and traits) constrained Multivariate Imputation Chained Equations procedure (MICE; Buuren and Groothuis‐Oudshoorn 2011). Although the number of species with missing information is high, the use of MICE has been shown to provide accurate trait estimates and conserve allometric relations when up to 60% of data is missing (Penone et al. 2014). The imputation process starts with an observed, incomplete data set for which a constrained multivariate distribution for the missing data is defined and then used to draw an imputation of the missing values using Markov Chain Monte Carlo techniques. A detailed description of the imputation approach, distribution of trait patterns for each replicate, and the description of the posterior distributions are presented in Appendix S2.

The imputation procedure was performed ten times, resulting in the same number of imputed versions of the database. To account for the variability in the imputation process, each of the imputed databases was used for the estimation of FD, resulting in ten values per AFE grid. Subsequent analyses, where we determined the effect of contemporary and historical environmental variables on FD were performed on each the FD imputed‐repetitions.

Two complementary multivariate FD metrics were used to describe the range (functional richness; *F*_Rich_) and variability (functional dispersion, *F*_Disp_) of the multidimensional functional space of each assemblage. *F*_Rich_ measures the trait spectrum range of the species assemblage, indicating the functional space occupied by a group of co‐occurring species regardless of their abundance (Mason et al. 2005). *F*_Disp_ measures mean multidimensional distance of individual species to the centroid of all co‐occurring species, providing a metric of both the packing of species with respect of the community “optimal” functional combination, and the functional segregation among coexisting species (Laliberté and Legendre 2010). Both *F*_Rich_ and *F*_Disp_ were estimated using only continuous traits. As evaluated traits have a lognormal distribution, these were log_10_ transformed and then normalized (mean = 0 and SD = 1) before the estimation FD. By doing this, we ensure that the measuring units or the shape of the distribution has an influence on the FD evaluation. As a complementary analysis and to determine which traits were the main predictors of changes in *F*_Rich_ and *F*_Disp_, the range (the difference between the minimum and maximum trait values) and dispersion (standard deviation) for each trait were estimated separately as univariate versions of these metrics.

### Environmental information

A total of 12 predictors, summarized as means for each AFE grid, which represents historical and contemporary environmental conditions, were used (units, estimation procedure, and sources described in Appendix S3). Selected predictors describe some of the most supported ecological mechanisms proposed as drivers of diversity patterns at large spatial scales (e.g., water–energy dynamics and habitat heterogeneity hypotheses; Ricklefs et al. 1993; Currie et al. 2004; Field et al. 2005; Kreft and Jetz 2007; Weiser et al. 2007; Kerkhoff et al. 2014). Spatially corrected pairwise correlations showed a weak association between predictors (90% of the correlations range between −0.25 and 0.25, with a maximum correlation of 0.55; Appendix S3). According to the criteria of Saatchi et al. (2007, 2011), such a low cross‐variable correlation implies that there is no multicollinearity in our predictors.

Historical predictors included climate velocity since the LGM and accessibility to LGM refugia. We use temperature and precipitation velocities since the LGM, as these provide a regional description of climatic stability in the region between the LGM (~21,000 years ago) and now. As in Sandel et al. (2011), climate velocities were estimated as the ratio of temporal trends (i.e., the absolute anomaly between the LGM and today) to spatial gradients (spatial dissimilarity in a 3 × 3 grid cell neighborhood) for a climatic variable. It is clear that as calculated here, climate velocity does not encapsulate the complex climatic dynamics during this period (i.e., changes during the Bølling–Allerød and Younger Dryas). Nevertheless, the strong correlations between LGM‐to‐present and 2000 years velocity estimates for the last 16,000 years (cf. Ordonez 2013) indicate that our long‐term estimation is a good representation of areas of climatic stability.

We use a distance‐based metric to determine the accessibility of a location from a region regarded as refugia during the LGM. Accessibility to LGM refugia was calculated for each AFE grid cell as the sum of inverse distances between an AFE grid cell and regions considered to be suitable for cool‐temperate trees during the LGM. Possible climatic refugia in Europe at the end of the LGM were estimated following Leroy and Arpe (2007) thresholds for European cold tolerant tree species (GDD ≥800°C, a mean temperature of the coldest month ≥15°C, and summer precipitation ≥50 mm). There is a strong association (*ρ *= 0.92; p_Dutilleul‐corrected_ = 0.001) between our climatically derived accessibility and the mean accessibility of 1016 European herbaceous and woody European plants estimated based on statistical models of LGM distribution (cf. Normand et al. 2011). Such a strong relation supports the use of our accessibility metric, as a descriptor of the potential colonization by a species from areas considered as LGM refugia regions.

Historical predictors were calculated based on 13 different climatic models providing predictions for the LGM. Climatic models come from the Palaeoclimate Modelling Intercomparison Project Phase III (PMIP_3_; http://pmip3.lsce.ipsl.fr/). A factor known to affect climate velocity estimates is the geographic resolution of the input data (Dobrowski et al. 2013). To avoid this problem, and match the resolution of contemporary variables, climatic models were statistically downscaled to a resolution of 10 × 10 arc‐min using standard change factor approach (Wilby et al. 2004). We combined the output from multiple climatic models as such an approach has been shown to provide a better description for climatic conditions at large scales (Doblas‐Reyes et al. 2000; Robertson et al. 2004). By combing all 13 models into a single map, we also reduced the possible effects of uncertainties and potential biases introduced by the specifications of the used climatic model. Using these averaged climate surfaces, we then estimated velocities and accessibility.

Contemporary predictors include variables related to the most supported hypothesis explaining both species diversity patterns (e.g., water–energy dynamics and habitat heterogeneity hypotheses; Weiser et al. 2007; Kerkhoff et al. 2014) and trait variability (Swenson et al. 2012a; Šímová et al. 2014) at broad geographic scales. Contemporary factors used to describe water–energy dynamics were absolute minimum temperature (estimated based on minimum temperature of the coldest month in Hijmans et al. 2005), number of growing degree‐days (5°C base temperature), thermal seasonality (intra‐annual temperature variability), total annual precipitation, water availability, and NDVI (Normalized Difference Vegetation Index). Predictors related to habitat heterogeneity included topographic heterogeneity represented by variability in elevation, number of major European soil types based on the Soil Atlas of Europe (defined according to the Food and Agriculture Organization World Reference Base for Soil Resources), and number of land use types). Annual precipitation, elevation heterogeneity, and glacial temperature and precipitation velocities exhibited a lognormal distribution and were log_10_‐transformed before the statistical analyses.

### The importance of historical and contemporary factors as predictors of functional diversity

The relation between environmental and FD estimates was determined using SAR_error_ models as these provide unbiased parameter estimates and have strong type I error control and good performance independently of the kind of spatial autocorrelation in the data (Kissling and Carl 2008). Spatial weights matrices in all SAR models were defined using the first neighbor of each grid cell. For each of the ten estimates of *F*_Rich_ and *F*_Disp_, we ran all 28,672 possible spatial autoregressive error regressions (SAR_error_) describing all possible noninteracting combinations among linear or unimodal (linear + quadratic) responses of all 12 historical and contemporary predictors evaluated in this study, for each imputed dataset.

For all 12 evaluated predictors, we determined its' relative support and importance using two approaches. First, we measured the explained variance of each contemporary and historical factor, using Nagelkerke (1991) pseudo‐*R* ^2^ values of single‐predictor models with a unimodal response (linear + quadratic terms). Secondly, we estimated variable relative support as *W*_AIC_ (Burnham and Anderson 2002), calculated by summing the *w*_AIC_ of all the models including the variable of interest. The same approach was used to determine the importance of range and dispersion of evaluated traits as *F*_Rich_ and *F*_Disp_ determinants, respectively (Appendix S5).

Using model‐averaged regression coefficients, we then measured the relative importance of historical variables as predictors of *F*_Rich_ and *F*_Disp_ while accounting for the effect of contemporary factors. For this, we averaged the standardized regression coefficients across all of the evaluated models, weighting each value by the *w*_AIC_ for the model that contained it. The use of model averaging based on *w*_*AIC*_ has received serious criticisms (Cade 2015), but multiple lines of evidence give us confidence in our estimates of variable support and averaged regression coefficients. First, there is no multicollinearity among the predictor variables. Second, the best fitting models consistently included similar variables. Third, the relative importance of variables was consistent across imputation datasets. Last, the explained variance of single‐predictor models supported the averaged model results.

To disentangle the effect of historical environmental conditions when all other evaluated variables are statistically controlled, we plotted the partial residuals for each historical predictor. Partial residual plots represent the relation between r + b * [historical factor] and a *historical factor*, where *r* is the residuals from the multiple‐predictor model that includes all the evaluated variables, and *b* is the regression coefficient estimate for the evaluated historical predictor from the same multiple‐predictor model.

As a supplementary analysis, we evaluated the relation between historical and contemporary predictors with SES (standardized effect sizes) for *F*_Rich_ and *F*_Disp_ as a way to determine species richness‐mediated effects on FD. This analysis was used to control for the possible confounding effects on local species richness on FD. For this, we used a null modeling approach where we generated 1000 estimates of *F*_Rich_ and *F*_Disp_ for each grid cell by randomizing the occurrence matrix, maintaining the original grid cell species richness. As in Swenson et al. (2012a), simulated estimates were used to estimate SES for *F*_Rich_ and *F*_Disp_ by subtracting the mean and dividing by the standard deviation of the null FD estimates. As spatial patterns and the relation to environmental conditions for SES (presented in Appendix S4) and unstandardized FD show similar patterns, our results and discussion are based on raw values only.

## Results

### Geographic patterns of European functional diversity

**Figure 1:**
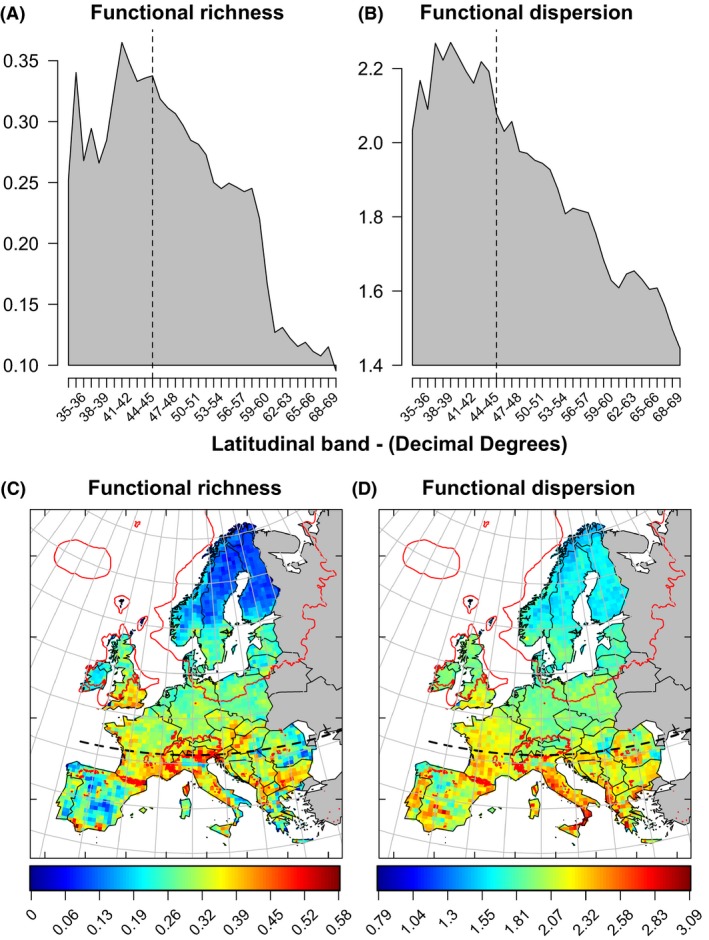
Functional diversity (FD) of European plants summarized by latitudinal bands (A, B) and for each of the Atlas Flora Europaeae grid cells (C, D). The dashed line in black in all panels represents the maximum northern limit of temperate tree full‐glacial refugia. Red delimited areas showed the maximum ice area present over 21,000 years ago (C, D). Represented values are the FD mean functional richness or dispersion, across the ten estimates calculated using ten distinct imputed trait datasets (see *Methods*).

Regions exhibiting high FD values were located primarily in areas south of the 46° latitude (dashed black line in Fig. 1A and B), which marks an estimate of the maximum northern limit of temperate tree full‐glacial refugia at the end of the LGM. Maximum *F*_Rich_ values were concentrated within the Mediterranean region, most notably in the southern mountainous regions (the Pyrenees, Apennines, Alps, and Dinaric Alps; Fig. 1C). Maximum *F*_Disp_ values likewise occurred in the Iberian, Italian, and Balkan peninsulas, notably along the Apennines and the southern Carpathians (Fig. 1D). These patterns were also observed for trait range and dispersion (Appendix S5). Multimodel inference showed a high support for trait range and dispersion of all evaluated traits as determinants of *F*_Rich_ and *F*_Disp_ (*W*_AIC_ **> 80% for all traits). However, the explained variance of single‐predictor models showed that seed mass and specific leaf area (SLA) range were the main drivers of *F*_Rich_; and trait dispersion in seed mass, specific leaf area and stem/wood density range determined variability in *F*_Disp_.

### Macro‐scale predictors of functional diversity

**Figure 2:**
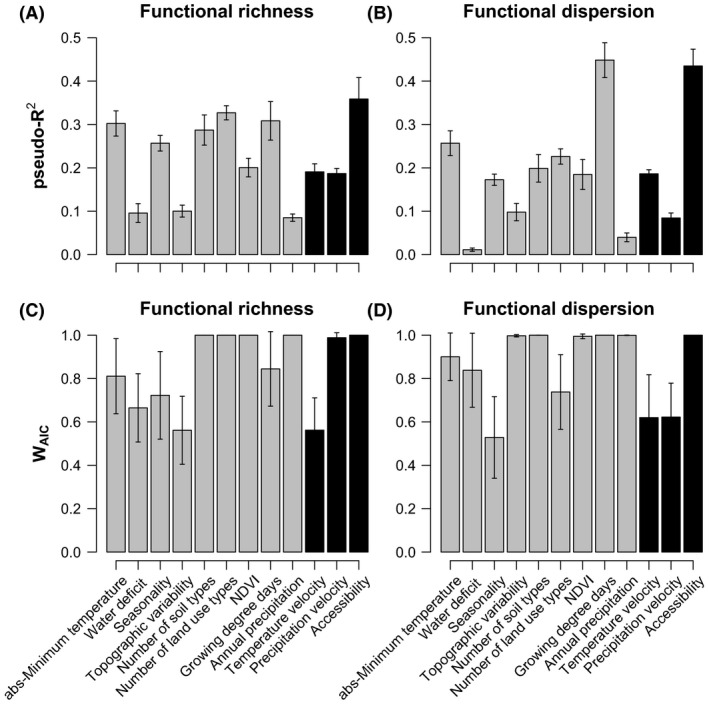
Explained variance (A, B) and relative support (C, D) of contemporary (gray) and historical (black) environmental predictors associated with realized functional richness and dispersion. The bar height and whiskers show the mean and 95%CI of the explained variance and relative importance score across the ten imputed datasets for each of the 12 evaluated predictors. Explained variance determined as Nagelkerke's (1991) pseudo‐*R* ^2^ values from single‐predictor models with a unimodal response (linear + quadratic terms). Relative support (*W*_AIC_) determined using Burnham and Anderson (2002) approach, where Akaike weights (*w*_AIC_) are summed across all models where the variable of interest was included as a linear or unimodal response. All relations between FD and environmental predictors determined using a spatial autoregressive error (SAR_error_) modeling approach (*see Methods*).

Comparing the explained variance of contemporary and historical environmental factors showed that accessibility, diversity of land uses, growing degree‐days, and absolute minimum temperature were the strongest individual predictors European plants *F*_Rich_ (Fig. 2A). In the case of *F*_Disp_, accessibility growing degree‐days and absolute minimum temperature were the strongest individual predictors according to the explained variance of single‐predictor models (Fig. 2B). By comparison, temperature and precipitation velocity have a moderate explanatory power (~20%; Fig. 2A and B) of *F*_Rich_ and *F*_Disp_ in the region.

Multimodel support of historical and contemporary environmental factors as predictors of European FD was comparable for both *F*_Rich_ and *F*_Disp_ (Fig. 2C and D). Based on summed Akaike weights (*W*_AIC_), accessibility and precipitation velocity had two of the highest relative importance scores of all 12 evaluated predictors for *F*_Rich_ (Fig. 2C). For *F*_Disp_, accessibility (Fig. 2D) showed one of the highest relative importance scores of the evaluated factors. The strong support of accessibility and precipitation velocity as predictors of European FD was supported by the ten imputed replicates. For *F*_Rich_, variation in the relative support of accessibility across imputed datasets ranged between 99% and 100% and of temperature velocity relative support fluctuated between 88% and 100%. In the case of *F*_Disp_, the relative support of accessibility across imputed datasets ranged between 99% and 100%.

Contemporary predictors with the highest relative importance scores changed between *F*_Rich_ and *F*_Disp_ (Fig. 2A vs. B). In the case of *F*_Rich_, contemporary predictors with the strongest multimodel support included annual precipitation (*W*_AIC_ 99%), NDVI (*W*_AIC_ 100%), the number of land uses (*W*_AIC_ 100%), and soil diversity (*W*_AIC_ 100%). Meanwhile, the contemporary predictors showing the highest multimodel support included for *F*_Disp_ were growing degree‐days (*W*_AIC_ 100%), soil diversity (*W*_AIC_ 99%), annual precipitation (*w*_AIC_ 99%), topographic variability (*W*_AIC_ 99%), and NDVI (*W*_AIC_ 99%).

**Figure 3:**
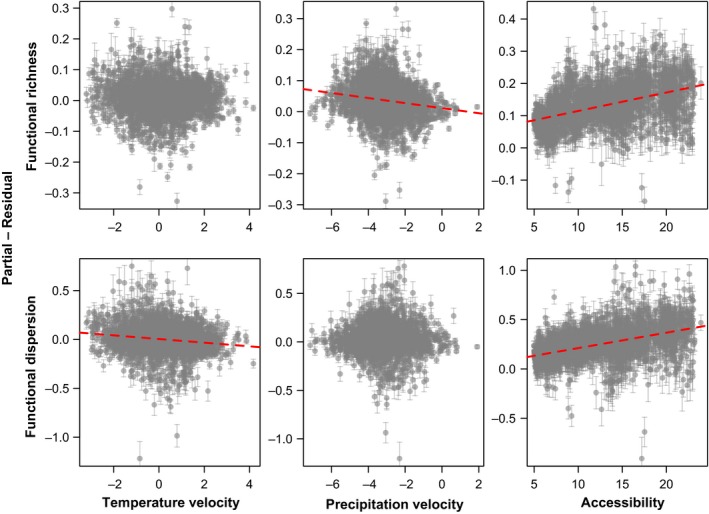
Partial regression plots representing the individual effect of climatic stability (measured as temperature and precipitation velocity) and accessibility to glacial refugia after all other variables in a multiple‐predictor model have been statistically controlled. Points represent the mean and whiskers represent the range of variation of partial residuals over the ten imputed datasets. Temperature and precipitation velocity measured as log_10_[km × decade^−1^], and accessibility in km^−1^. Only the regression lines of significant associations are plotted (red dashed lines).

After controlling for other variables, *F*_Rich_ and *F*_Disp_ decreased as climatic velocities increased, and accessibility to LGM refugia declined (Fig. 3). Accessibility to LGM refugia proved to be a significant predictor of both *F*_Rich_ and *F*_Disp_ in the region. In the case of climatic stability, precipitation velocity proved to be a major determinant of *F*_Rich_, and temperature velocity proved to be a major determinant of *F*_Disp_. We consider that the importance of historical predictors, even after the effect of contemporary factors has been controlled, provides robust and conservative evidence for the prevalence of legacies of late‐Quaternary environmental changes in European FD patterns.

Model‐averaged standardized coefficients (Table 1) provided additional support for the importance of accessibility as a predictor of *F*_Rich_ and *F*_Disp_, and precipitation velocity as a predictor of *F*_Rich_. In the case of *F*_Rich_, accessibility to LGM refugia has been particularly influential in shaping the observed geographic patterns, in combination with contemporary factors such as the number of land uses, growing degree‐days, soil diversity, absolute minimum winter temperature, and seasonality (Table 1). In the case of *F*_Disp_, accessibility to LGM refugia acted in conjunction with growing degree‐days, annual precipitation, absolute minimum winter temperature, and NDVI (Table 1).

**Table 1:** Model‐averaged standardized regression coefficients for contemporary and historical predictors as predictors of *F*_Rich_ and *F*_Disp_ for European plants in the Atlas Florae Europaeae. Coefficients were summarized using Burnham and Anderson's (2002) model averaging approach and indicate the *w*_*AIC*_‐weighted mean of model‐averaged regression coefficients across all imputed databases. Variables with significant support (*W*_AIC_ ≥ 0.8) in bold. b: linear response. b^2^: quadratic response. Empty cells indicate that model‐averaged standardized coefficients were lower than 0.0005

	Functional richness		Functional dispersion	
	b	b^2^	b	b^2^
Contemporary predictors				
Absolute minimum winter temperature	−**0.113**		−**0.242**	**0.21**
Water balance	−0.053	−0.001	−**0.15**	−**0.044**
Seasonality	0.216	−0.22	−0.02	
Topographic heterogeneity	0.058	0.034	**0.121**	
No of soil types	**0.143**		**0.159**	−**0.046**
No of land uses	**0.216**	**0.023**	−0.034	
NDVI	**0.17**		**0.187**	
Growing degree‐days	−**0.154**		**0.356**	
Annual precipitation	**0.081**		**0.279**	−**0.122**
Historical predictors				
Temperature velocity	−0.009	0.007	−0.03	
Precipitation velocity	−**0.097**		0.004	
Accessibility	**0.968**	−**0.612**	**0.285**	

## Discussion

Both the geographic patterns in FD and the relative importance of historical predictors indicated that the imprints of environmental changes during the late Quaternary could still be detected in current FD across Europe. Maximum FD values were found south of the 46°N latitude, areas considered as refugia for nonboreal temperate species during the LGM (Bennett et al. 1991; Leroy and Arpe 2007; Abellán and Svenning 2014). These geographic patterns were consistent with the climatic and colonization history of northern Europe over the last 21,000 years (Bennett et al. 1991; Ehlers and Gibbard 2004). They are also consistent with the restriction of Mediterranean and multiple temperate species to glacial refugia in southern (Leroy and Arpe 2007; Normand et al. 2011) and eastern Europe (Svenning and Skov 2004; Willis and van Andel 2004).

Our continental‐level assessment of historical climatic effects on European FD found strong individual influences of historical conditions, which are comparable in importance and magnitude to those of contemporary conditions. Such pattern highlights the mutual importance and combined effects of historical and contemporary conditions in determining regional FD geographic trends. The directions of the associations between realized FD metrics and historical factors were consistent with the hypothesis of increasing *F*_Rich_ and *F*_Disp_ with proximity to LGM refugia and slower climate velocities since the LGM.

Like most biogeographical studies linking past or present environmental conditions to diversity patterns, our study lacks mechanistic detail. Nonetheless, geographic patterns of dispersal traits and the extensive literature on the climatic and floristic history of Europe provide important clues into which mechanism are the most likely drivers behind the importance of late‐Quaternary climate change as a predictor of current FD. For example, seed mass showed a south‐to‐north pattern geographic pattern, and the range and dispersion of this trait was an important factor shaping European *F*_Rich_ and *F*_Disp_ patterns. These two conditions indicate how dispersal‐limited recolonization dynamics of climatically suitable areas can potentially be one of the most signifi important mechanisms shaping the effects of historical environmental factors on contemporary FD, via changes in species distribution ranges (Normand et al. 2011). Moreover, dispersal‐limited re‐colonization can interact with regional geographical fragmentation in the species pools resulting from physical dispersals barriers such as sea straits and mountain ranges (Nekola and White 1999), strengthening the importance of historical predictors such accessibility to LGM refugia.

The cumulative historical environmental filtering of unsuitable phenotypes from the species pool can also impose lasting impact on FD patterns. Two mechanisms might explain how the environmental filtering could leave lasting imprints on local diversity. First, nonrandom extinctions may be driven by changes in climatic conditions since the LGM (Svenning 2003). Second, regional species pools being determined via the historical filtering of species according to traits related to physiological or ecological tolerances (Mouillot et al. 2013; Zanne et al. 2013; Nogués‐Bravo et al. 2014; Eiserhardt et al. 2015). The effect of these two mechanisms would change across a region, in response to the geography of historical changes in climatic conditions and modifications in the species assemblages. Although we cannot provide direct evidence for this, the deficits in species richness (Svenning et al. 2010; Ronk et al. 2015) and FD (Ordonez and Svenning 2015) reported for European plants, indicate the relevance of this mechanism as a determinant as a driver of reported importance of historical environmental conditions.

Apart from the relations of historical drivers to *F*_Rich_ and *F*_Disp_, there were also strong links to current climate and environment. As expected, contemporary mild climatic extremes and high levels of environmental heterogeneity exhibited positive effects on FD, but the strength of these effects varied between *F*_Rich_ and *F*_Disp_. The influence of absolute minimum winter temperature, annual precipitation, and environmental heterogeneity have on *F*_Rich_ indicates how water‐energy availability and contemporary environmental variability determine the potential size of the functional space an assembly can occupy. In comparison, the association of *F*_Disp_ with absolute minimum winter temperature, NDVI, growing degree‐days, and annual precipitation is consistent with increasing levels of trait divergence in areas where temperature and water extremes filters out species with unsuitable traits.

The influence of contemporary predictors on FD illustrates the well‐known effects of recent environmental conditions as key predictors of species distribution and richness patterns among plants (Currie et al. 2004; Field et al. 2005; Kreft and Jetz 2007; Swenson et al. 2012b). Nevertheless, our results show a comparable explained variance and importance of historical factors, when compared to contemporary conditions as predictors of European *F*_Rich_ and *F*_Disp_. Moreover, the high multimodel support for historical and contemporary environmental conditions, the consistent high effect of both set of factors (measured as model‐averaged regression), and the fact that historical and contemporary predictors determine FD support or hypothesis that the legacies of Late‐Quaternary climate changes can still be seen today.

Two major caveats can potentially bias our assessment of the importance of historical environmental conditions as predictors of European FD patterns. First, the AFE has a strong taxonomic bias, with groups such as Brassicaceae, Caryophyllaceae, and Ranunculales constituting the majority of the species in the database. However, by comparing the estimates of FD of phylogenetic‐independent groups with a pan‐European distribution (Appendix S1), we show that there is a generalized geographic pattern in the functional space size and dispersion of European plants. Such a consistency across groups with an independent evolutionary history gives us confidence that the reported FD geographic patterns are robust to any AFE taxonomic biases, as also shown when comparing AFE and plot‐based species richness patterns in Europe (Ronk et al. 2015).

Second, climatic reconstructions based on climate models have been shown to large uncertainties, which result in differences in the ability of climatic reconstructions to describe local and regional climatic dynamics. To prevent this issue, we use a multimodel ensemble to describe millennial temperature and precipitation patterns at the end of the LGM. The use of multimodel ensemble is a common practice in climatology when the goal is describing geographic patterns of climate (Doblas‐Reyes et al. 2000; Robertson et al. 2004). Although these estimates are better in representing historical climates, they still have a greater uncertainty than estimates of present‐day environmental factors. However, given that historical predictors were among the factors with largest model support, explanatory power, and effects as predictors of European FD, we are confident in our estimates of late‐Quaternary historical imprints on contemporary European‐FD.

## Conclusion

In this study, we have shown that historical factors are as important as contemporary environmental conditions in predicting FD patterns across Europe. The importance of historical environmental conditions indicates that European plant FD is related to environmental‐driven dynamics across the last 21,000 years. The importance of accessibility and climate velocity as predictors of FD has important implication when determining the extent to which historical climatic stability and accessibility to climatic refugia can affect FD.

The traits evaluated in this study are related to the way species assemblages accumulate and cycle biomass and nutrients in the environment, linking the recorded influence of historical conditions to changes in ecosystem functioning (i.e., productivity and nutrient cycling). Consequently, our results indicate that the importance of historical environmental factors would most likely percolate to the ecosystem functions performed by European plant assemblages. If this is the case, future climate change will not only elicit short‐term shifts in ecosystem functioning, but also induce long‐term impacts on ecosystem functioning. Incorporating the underlying mechanisms behind the importance of historical environmental conditions into predictive models for future vegetation‐related ecosystem functioning will enhance the accuracy of climate change impact and dynamics estimations.

## Conflict of Interest

None declared.

## Supporting information


**Appendix S1.** Robustness of functional diversity estimates to the taxonomic coverage.
**Appendix S2.** Traits sources and description of the trait imputation procedure.
**Appendix S3.** Geographic patterns and association between contemporary and historical environmental variables.
**Appendix S4.** Individual macro‐scale determinants of species richness standardized functional richness and dispersion.
**Appendix S5.** Association between univariate (trait range and dispersion) and multivariate metrics of functional diversity.
