# Supplementary material for: Strong paleoclimatic legacies in current plant functional diversity patterns across Europe
Source: Ecol Evol. 2016 Apr 18;6(10):3405–16. doi: 10.1002/ece3.2131 (PMC4870221; doi:10.1002/ece3.2131)
Supplement: Supplementary file 1 — Appendix S1. Robustness of functional diversity estimates to the taxonomic coverage. Appendix S2. Traits sources and description of the trait imputation procedure. Appendix S3. Geographic patterns and association between contemporary and historical environmental variables. Appendix S4. Individual macro‐scale determinants of species richness standardized functional richness and dispersion. Appendix S5. Association between univariate (trait range and dispersion) and multivariate metrics of functional diversity. [file ECE3-6-3405-s001.docx]

Supporting Information

*Strong paleoclimatic legacies in current plant functional diversity patterns across Europe*

*Alejandro Ordonez^1^ and Jens-Christian Svenning^1^*

***^1^****Section for Ecoinformatics and Biodiversity, Department of Bioscience, Aarhus University, Ny Munkegade 114, DK-8000 Aarhus C, Denmark*

***Supporting Information Overview***

This document provides supplementary information not provided in the main text of the article “*Strong paleoclimatic legacies in current plant functional diversity patterns across Europe”*.

***Appendix S1*** Robustness of functional diversity estimates to the taxonomic coverage.

***Appendix S2*** Traits sources and description of the trait imputation procedure.

***Appendix S3*** Geographic patterns and association between contemporary and historical environmental variables.

***Appendix S4*** Individual macro-scale determinants of species richness standardized functional richness and dispersion.

***Appendix S5*** Association between univariate (trait range and dispersion) and multivariate metrics of functional diversity.

***Appendix S1*** Robustness of functional diversity estimates to the taxonomic coverage*.*

We determined the consistency of the geographic patterns of functional richness and dispersion estimates by comparing estimates based on all the species in the database and estimations using only orders with a pan-European distribution and highest species richness: Brassicales, Caryophyllales, Fagales, Malpighiales, Ranunculales, Rosales, Saxifragales.

| 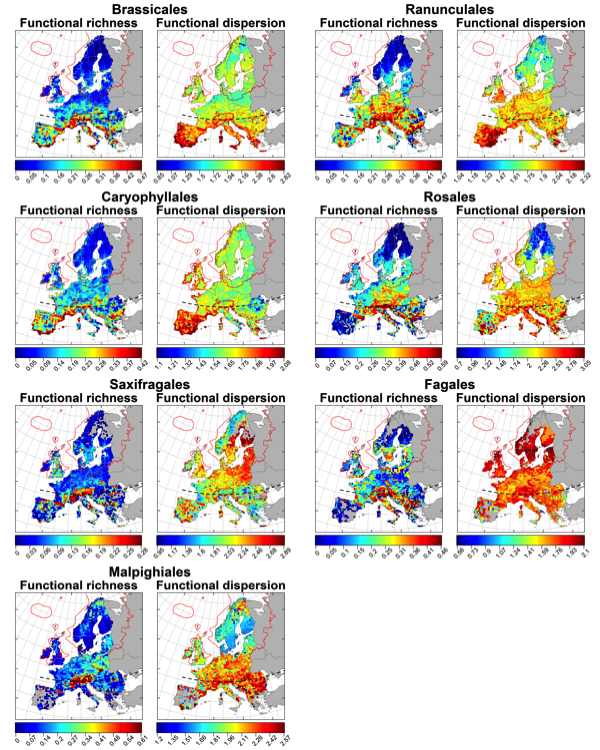 |
| --- |
| **Figure S1. Functional richness and dispersion for all species within a pan-European Order.** Maps show the mean over ten estimates of functional diversity for each of the Atlas Flora Europaeae grid cells (~50 km × 50 km). Evaluated orders are Brassicales, Caryophyllales, Fagales, Malpighiales, Ranunculales, Rosales, and Saxifragales. The black dashed line signals the 46° N latitude, which marks an estimate of the maximum northern limit of temperate tree full glacial-refugia. Red delimited areas show the maximum extent of the ice-sheet ~21,000 years ago. |

As expected, functional diversity magnitude changed across evaluated orders with pan-European coverage (**Figure S1**). However, the relation between functional diversity and with latitude was consistent across evaluated orders, with functional richness and dispersion increasing in a north-to-south direction. The consistency of this pattern is highlighted by the uniformity in the relation between single order estimates those using all species (**Figure S2**).

| ****** |
| --- |
| **Figure S2. Spearman rank correlations coefficients estimates of functional (A) richness and (B) dispersion** among the nine orders with continental level coverage, and between these with estimates of functional richness and dispersion using all species. |

We also evaluated the sensitivity of our FD estimates to the omission of widely distributed and functionally important taxonomic groups. For this, we estimated F_Rich_ and F_Disp_ using a jack-knife approach, in which we left out all species from a given order. The analyses focused on the orders with the highest species richness (Brassicales, Caryophyllales, Fagales, Malpighiales, Piperales, Ranunculales, Rosales, Santalales, Saxifragales). Second, we compared F_Rich_ and F_Disp_ estimates excluding species classified as introduced aliens in Europe to those using all the species. Convergence in the observed patterns would indicate that the observed geographic FD patterns are robust to the taxonomic coverage and recent introductions in the region.

| 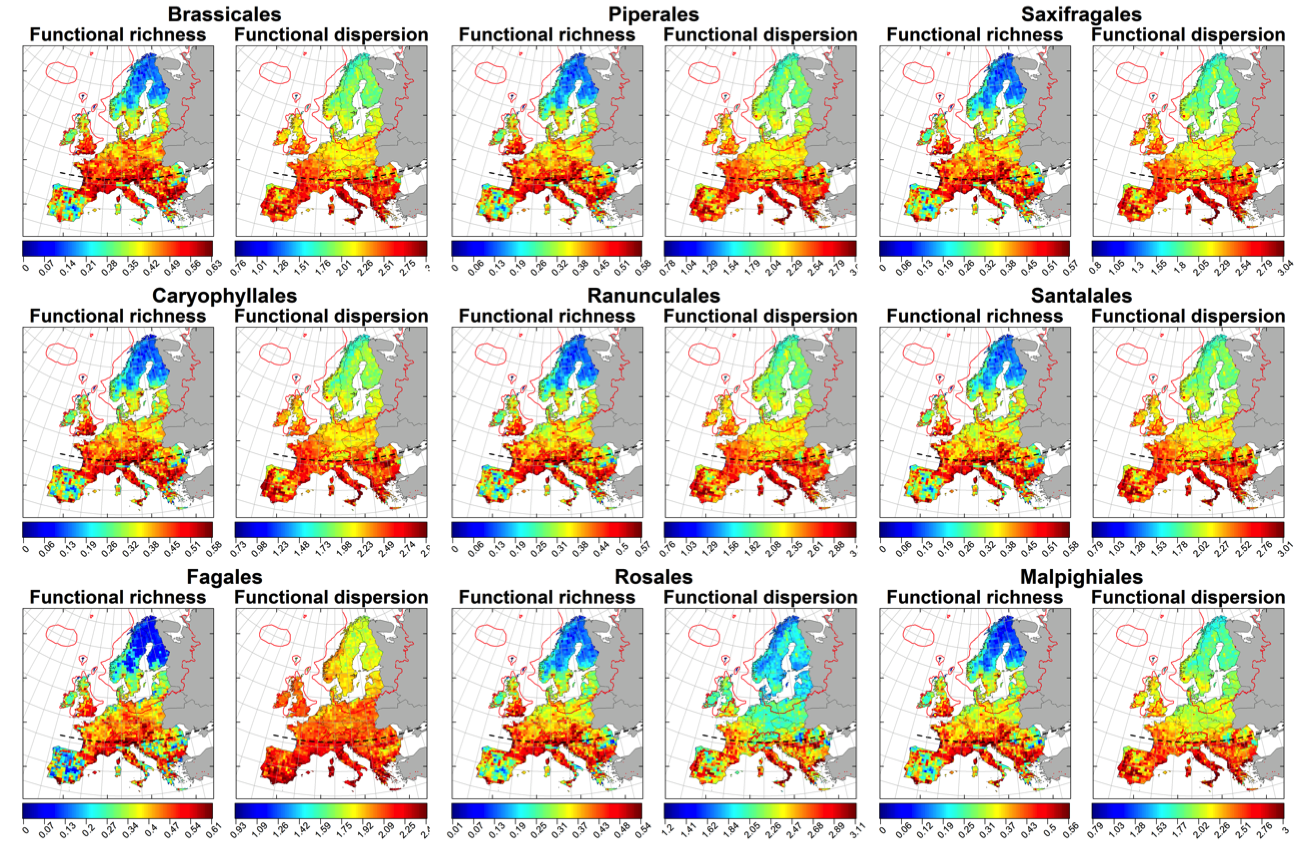 |
| --- |
| \| **Figure S3. Functional richness and dispersion of European assemblies after removing all the species within a pan-European Orders**. Plant orders were removed sequentially across the nine orders with the highest richness and the best geographical coverage for europe. Evaluated orders and figure elements as in **Figure S1**. \| \| --- \| |

Removing all species from a widely distributed and species-rich order did not significantly change F_Rich_ and F_Disp_ geographic patterns (**Figure S3**). The tight correlation between order-omitted and all species FD (ρ ranged between 0.964 and 1 for F_Rich_ and 0.893, and 1 for F_Disp_; with pDutilleul-corrected≤0.001 in all cases) estimates show the robustness of our F_Rich_ and F_Disp_ estimates to the pool of species used for the analysis. Similarly, contrasts of FD estimates including and excluding plants alien to Europe show a tight correlation (F_Rich_ ρ: 1 and F_Disp_ ρ: 0.945; with p_Dutilleul-corrected_≤0.001 in both cases) indicating that including naturalized alien species or agriophytes does not significantly affect our FD estimates.

***Appendix S2*** Traits sources and description of the trait imputation procedure.

***Used traits –*** The analysis in this work are based on five ecomorphological traits: specific leaf area (SLA, cm^2^*g^-1^), seed mass (mg), maximum stem height (m), stem/wood density (kg*m^-3^) and growth form. These represent a portion of the best traits for predicting the geographic distribution of vegetation types and assessing the mean plant physiological response in a region. Furthermore, these traits have been used to discriminate between distinctive functional strategies among concurring plants ([Westoby *et al.* 2002](#_ENREF_38); [Falster & Westoby 2003](#_ENREF_11); [Wright *et al.* 2004](#_ENREF_39); [Chave *et al.* 2009](#_ENREF_8)).

The SLA of a plant indicates how much light capturing area a plant produces for each gram of leaf tissue, hence reflecting the positioning along the leaf economics spectrum ([Wright *et al.* 2004](#_ENREF_39)). SLA is negatively correlated with leaf thickness, lifespan, toughness and sensitivity to herbivory and positively correlated with mass-based measurements of leaf nitrogen content, photosynthetic capacity, plant relative growth rate, all of which are relevant to plant performance ([Wright *et al.* 2004](#_ENREF_39)). H_max_ represents the balance between the gains from access to light, the cost of structural support (given the disturbance regime), water transport and sensitivity to biomass loss from mechanical disturbances. H_max_ is also a proxy for other essential traits indicating plant growth ([Falster & Westoby 2003](#_ENREF_11)). SWT represents the balance between the number and size of offspring, which is often related to age-dependent survival probability ([Westoby *et al.* 2002](#_ENREF_38)). Furthermore, SWT is often negatively correlated with dispersal distance (in the case of wind-dispersed species) and positively correlated with seedling survival probability under light or water-limited conditions ([Leishman *et al.* 2000](#_ENREF_20); [Moles & Westoby 2006](#_ENREF_24)). Lastly, wood density represents the tradeoffs between growth rates, construction costs and mortality rates ([wood economics spectrum; Chave *et al.* 2009](#_ENREF_8)). Within this spectrum, a species will fall along a continuum between high volumetric growth rates, low construction costs, and high mortality rates vs. low volumetric growth rates, high construction costs, and low mortality rates ([Swenson & Enquist 2007](#_ENREF_34); [Chave *et al.* 2009](#_ENREF_8)).

**Table S1.** Trait information sources

| **Trait** | **Source** |
| --- | --- |
| Specific leaf area (SLA) | [Wright *et al.* (2004](#_ENREF_39)); [Kleyer *et al.* (2008](#_ENREF_19)); [Ordonez *et al.* (2010](#_ENREF_28)); [Kattge *et al.* (2011](#_ENREF_18)); [Ordonez and Olff (2013](#_ENREF_27)) |
| Seed mass  (SWT) | [Moles and Westoby (2006](#_ENREF_24)); [Kleyer *et al.* (2008](#_ENREF_19)); [Liu *et al.* (2008](#_ENREF_22)); [Ordonez *et al.* (2010](#_ENREF_28)); [Ordonez and Olff (2013](#_ENREF_27)) |
| Maximum stem height (H_max_) | [Kleyer *et al.* (2008](#_ENREF_19)); [Ordonez *et al.* (2010](#_ENREF_28)); [Kattge *et al.* (2011](#_ENREF_18)); [Ordonez and Olff (2013](#_ENREF_27)) |
| Wood density  (WD) | [Kleyer *et al.* (2008](#_ENREF_19)); [Chave *et al.* (2009](#_ENREF_8)) |
| Growth form  (GF) | [Kleyer *et al.* (2008](#_ENREF_19)); [Kattge *et al.* (2011](#_ENREF_18)); [USDA-NRCS (2013](#_ENREF_37)) |

Mean trait values were determined initially from multiple databases (**Table S1**). Remaining gaps in the database (~50% of species in the database per trait) were filled in using the Multivariate Imputation Chained Equations procedure ([Buuren & Groothuis-Oudshoorn 2011](#_ENREF_7)). In brief, the imputation procedure involved specifying a multivariate distribution for the missing data and drawing imputation from the joint conditional distribution using Markov Chain Monte Carlo (MCMC) techniques (see below for an expanded explanation).

***Trait imputation*** – Different methods can be used to “*filling in*" the missing data. For example, taxonomic imputation ([Baker *et al.* 2004](#_ENREF_2); [Malhi *et al.* 2006](#_ENREF_23); [Baker *et al.* 2009](#_ENREF_1); [Shan *et al.* 2012](#_ENREF_33)), ancestral state reconstruction ([Bruggeman *et al.* 2009](#_ENREF_3)), phylogenetic Eigen-vectors ([Diniz *et al.* 2014](#_ENREF_9)), phylogenetic generalized least squares ([Swenson 2014](#_ENREF_35)), or multiple imputation (MI hereafter; [Schafer 1999](#_ENREF_31); [Rubin 2009](#_ENREF_29)). This last one is the method of choice for complex, incomplete data problems such as sparse data matrices, when no fully resolved and dated phylogeny is available. MI is a Monte Carlo technique in which the missing values are replaced by a small set of simulated versions (usually <10). MI imputed-data estimates for missing values are expected to give reasonable predictions for the mean missing data while the variability among them reflect both the data and the imputation uncertainty. Also, MI is often preferred over other imputation techniques as it is readily available, provides an approximate solution, is not problem-specific, does not rely on phylogenetic hypotheses of relatedness, and perhaps more importantly, is not complicated to implement.

The MI implementation involves specifying a multivariate distribution for the missing data, and drawing imputation from their conditional distributions by Markov Chain Monte Carlo (MCMC) and a Gibbs sampler techniques (an approach that is valid if a multivariate distribution is a reasonable description of the data as in the case of trait values). In those cases where it is not possible to specify a multivariate distribution for the missing data, an MI can be implemented on a variable-by-variable basis by a set of conditional densities, one for each incomplete variable (an approach that is valid if the imputed values are orthogonal to each other), and then draw imputations by iterating over the conditional density (sampling the assumed density).

The starting point of the imputation process is defining if the data is missing at random or not. In our case, it is somehow reasonable to make this assumption given the structure of the dataset, and the fact that it there is no *a priori* reason to expect that certain groups are systematically missing measurements. The next step is selecting the imputation model, which is nothing more than determining how the missing value multivariate distribution will be generated. This implies choosing (1) the scale of the variable to be imputed, (2) the variables used for the imputation and the relation between these, (3) order in which variables should be imputed and (4) the number of iterations and how many imputed values are generated (low iteration lead to no convergence in the of the Gibbs sampler, and low imputed values to a under-coverage of the distribution).

We used the fastest imputation methods implemented in *mice*: Predictive mean matching – (hereafter *pmm*). The *pmm* approach is similar to a regression method, except that for each missing value, it imputes a value randomly from a set of observed values whose predicted values are closest to the predicted value for the missing value from the simulated regression model ([Heitjan & Little 1991](#_ENREF_14); [Schenker & Taylor 1996](#_ENREF_32)). The predictive mean matching method requires the number of closest observations to be specified. A smaller number of observations tend to increase the correlation among the multiple imputations for the missing observation and results in a higher variability of point estimators in repeated sampling. pmm is a data-intensive method, requiring a large number of observations for it to work. The predictive mean matching method ensures that imputed values are plausible and might be more appropriate than the regression method if the normality assumption is violated ([Horton & Lipsitz 2001](#_ENREF_16)).

We did our imputations on a dataset consisting of traits (SLA- H_max_ -SWT-WD) recorded on a continuous scale (where the observations are missing), and two categorical variables Genus and growth forms (Ferns, Forb/herb, Graminoid, Shrub, Tree, Vine) for which information is available for all the evaluated species. When performing the imputation, all the attributes in the input matrix were used to generate the multivariate distribution/model to define the possible values of empty cells, but only imputed values for SLA- H_max_ -SWT-WD. By including a species genus as a constraint, we allow a simple phylogenetic constraint to the imputation procedure. Also, the inclusion of growth form as a second categorical factor further restricts the pool of species from which imputed traits are drawn. The imputation procedure consisted of 10 imputed values for each empty cell, generated from 1000 iterations per imputation. As the order of imputation matters when generating the multivariate distribution, traits were imputed in decreasing amount of messiness (H_max_-SWT-SLA-WD). R package mice ([Buuren & Groothuis-Oudshoorn 2011](#_ENREF_7)) was used to generate imputed values.

| 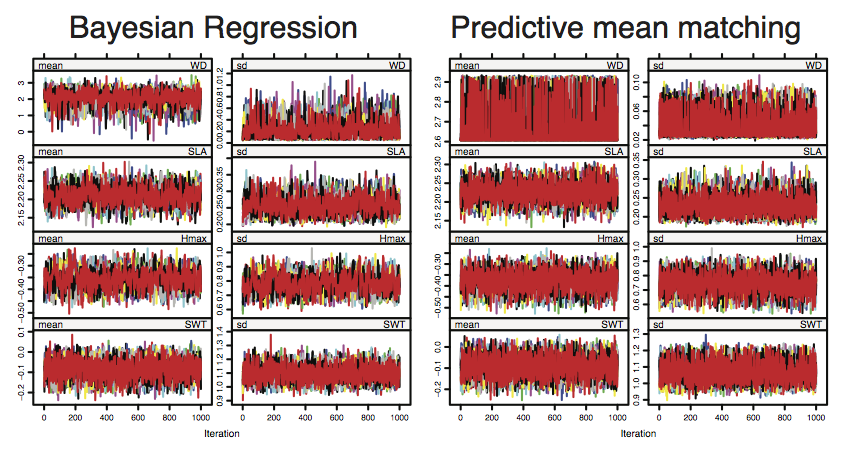 | **Figure S4.** **Gibbs sampler mixture for the imputed traits.** Each series of plots represents replicates for a Predictive mean matching imputation approaches. SLA: specific leaf area, SWT: Seed mass, H_max_: maximum stem height and WD: wood or stem density. |
| --- | --- |

|  | |
| --- | --- |
| 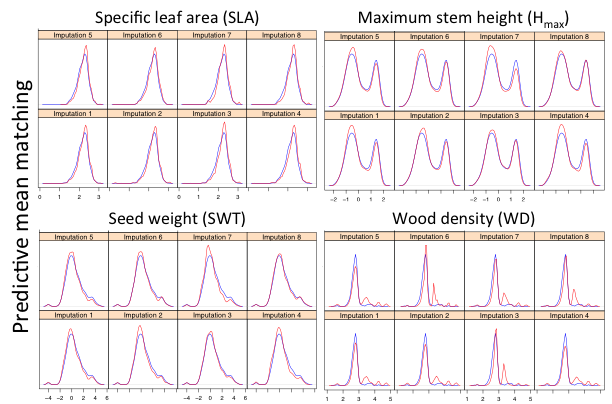 | **Figure S5** **Kernel density estimates for the marginal distributions of the observed and imputed trait values** based on a predictive mean matching approach (*pmm*). Each series of plots represents each model configurations for each evaluated traits. Red line display observed values and blue lines show imputed values |

The imputation procedure showed good mixtures for the Gibbs sampler (the imputed values are not fixed at the initial imputation and show good mixing, **Figure S4**). Moreover, kernel density estimates for the marginal distributions of the observed and imputed trait values shows that the imputed values are in the same ballpark than observed values (e.g., marginal posterior distributions all almost one on top of the other, **Figure S5**) for SLA, SWT, and H_max_. In the case of WD, imputed values are lower than the observed values, and marginal distributions do not overlap (perhaps because it is estimating WD for non-woody plants).

***Appendix S3*** Geographic patterns and association between contemporary and historical environmental variables

A total of 12 variables were used to summarize historical climatic stability gradients, accessibility to refugia at the end of the last glacial maximum (LGM), and multiple aspects of the contemporary environment, namely climate soils and land use (**Table S1** and **Figure S7**). Selected variables are close related to some of the ecological mechanisms proposed to drive diversity patterns at continental and global scales.

| **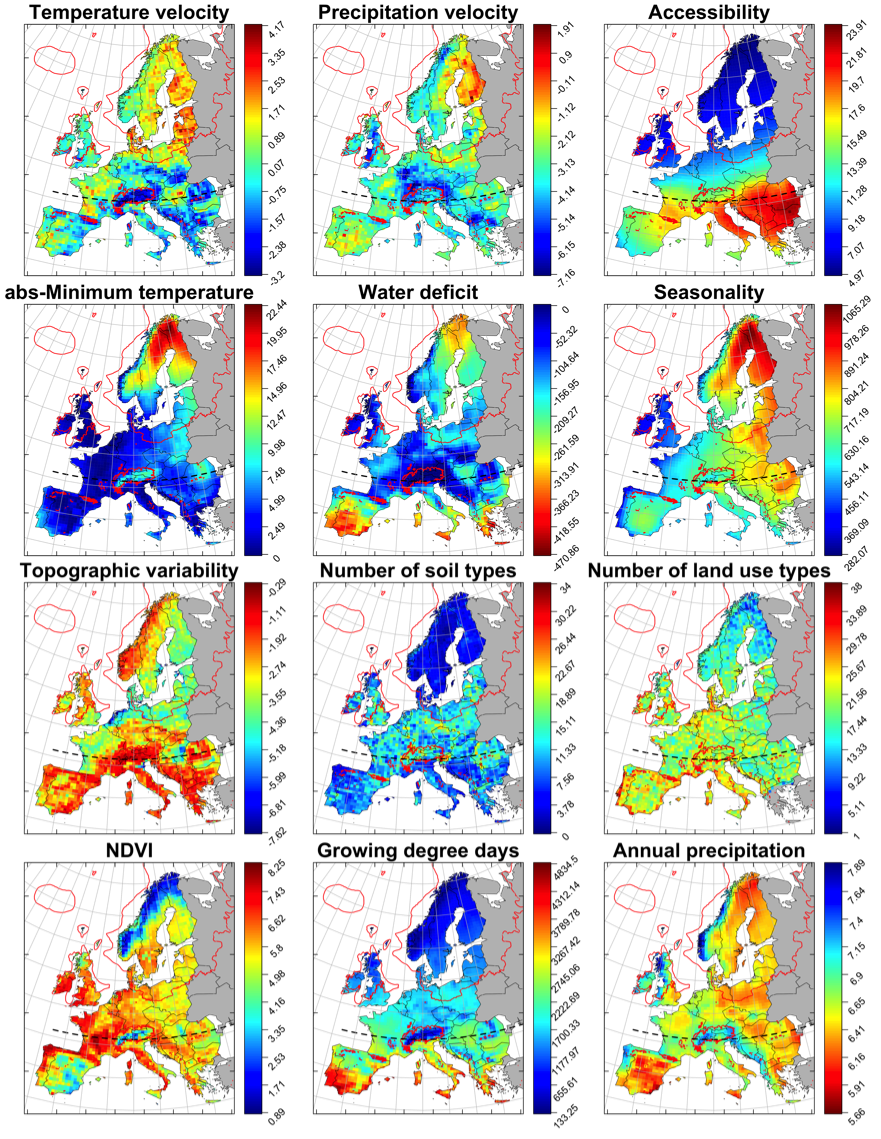** |
| --- |
| **Figure S7. Contemporary and historical environmental predictors.** |

**Table S1.** Historical and contemporary predictor variables used to explain spatial variation in European functional richness and dispersion. All variables were summarized for AFE grid cell (~50x50km).

| **Predictor variables**  **(Units)** | **Estimation - Data source** | | |
| --- | --- | --- | --- |
| Mean annual temperature velocity – [LGM to present]  (km*decade^-1^) | Calculated using ([Sandel *et al.* 2011](#_ENREF_30)) method for each of 13 models in the Paleoclimatic Modelling Intercomparison Project Phase III (PMIP_3_; http://pmip3.lsce.ipsl.fr/) with data for the LGM. Used values are the mean across all 13 models | | |
| Annual precipitation velocity– [LGM to present]  (km*decade^-1^) | Calculated using ([Sandel *et al.* 2011](#_ENREF_30)) method for each of 13 models in the Paleoclimatic Modelling Intercomparison Project Phase III (PMIP_3_; http://pmip3.lsce.ipsl.fr/) with data for the LGM. Used values are the mean across all 13 models | | |
| Accessibility to postglacial re-colonization from LGM refugia  (km^-1^) | Calculated for a location as the summed inverse distance of each focal to all cells considered as refugia during the LGM. Refugia regions established for the LGM predictions of 13 models in the Paleoclimatic Modelling Intercomparison Project Phase III (PMIP_3_; <http://pmip3.lsce.ipsl.fr/>) based on ([Leroy & Arpe 2007](#_ENREF_21)) model of minimum climatic requirements for cool-temperate trees. Used values are the mean across all 13 models | | |
| Absolute minimum temperature  (abs °C) | Estimated from monthly values using the  Worldclim dataset ([Hijmans *et al.* 2005](#_ENREF_15)) | | |
| Growing degree-days  (°C) | Estimated based on 5°C reference temperature, using monthly values in the Worldclim dataset ([Hijmans *et al.* 2005](#_ENREF_15)) and approach. | | |
| Intra-annual temperature variability  (mm* yr^-1^) | Standard deviation of monthly temperature values as estimated in the Worldclim dataset ([Hijmans *et al.* 2005](#_ENREF_15)) | | |
| Total annual precipitation | Summed total monthly precipitation, estimated based on monthly values in the Worldclim dataset ([Hijmans *et al.* 2005](#_ENREF_15)) | | |
| Water availability  (mm* yr^-1^) | Calculated following (Ohlemüller *et al.* 2006) as the summed difference between monthly precipitation ([obtained from the Worldclim dataset Hijmans *et al.* 2005](#_ENREF_15)) and monthly potential evapotranspiration (estimated using Thornthwaite equation ([Thornthwaite 1948](#_ENREF_36)) based on Worldclim ([Hijmans *et al.* 2005](#_ENREF_15)) monthly temperature and ([Forsythe *et al.* 1995](#_ENREF_13)) day length approximation. | | |
| Normalized Difference Vegetation Index [NDVI]  (Unitless) | Mean yearly NDVI over the 1981 to 2003 period based on the FAO Annual Sum NDVI ([FAO 2014](#_ENREF_12)). | | |
| Elevation variability  (m) | Standard deviation of elevation base on the on map of Worldclim dataset ([Hijmans *et al.* 2005](#_ENREF_15)) | | |
| Number of soil types  (Count) | Number of major European soil types based on the Soil Atlas of Europe ([Jones *et al.* 2005](#_ENREF_17)). | | |
| Number of Land use types  (Count) | Number of land use types derived from the CORINE Land Cover map of Europe ([Büttner *et al.* 2004](#_ENREF_6)). | | |
| 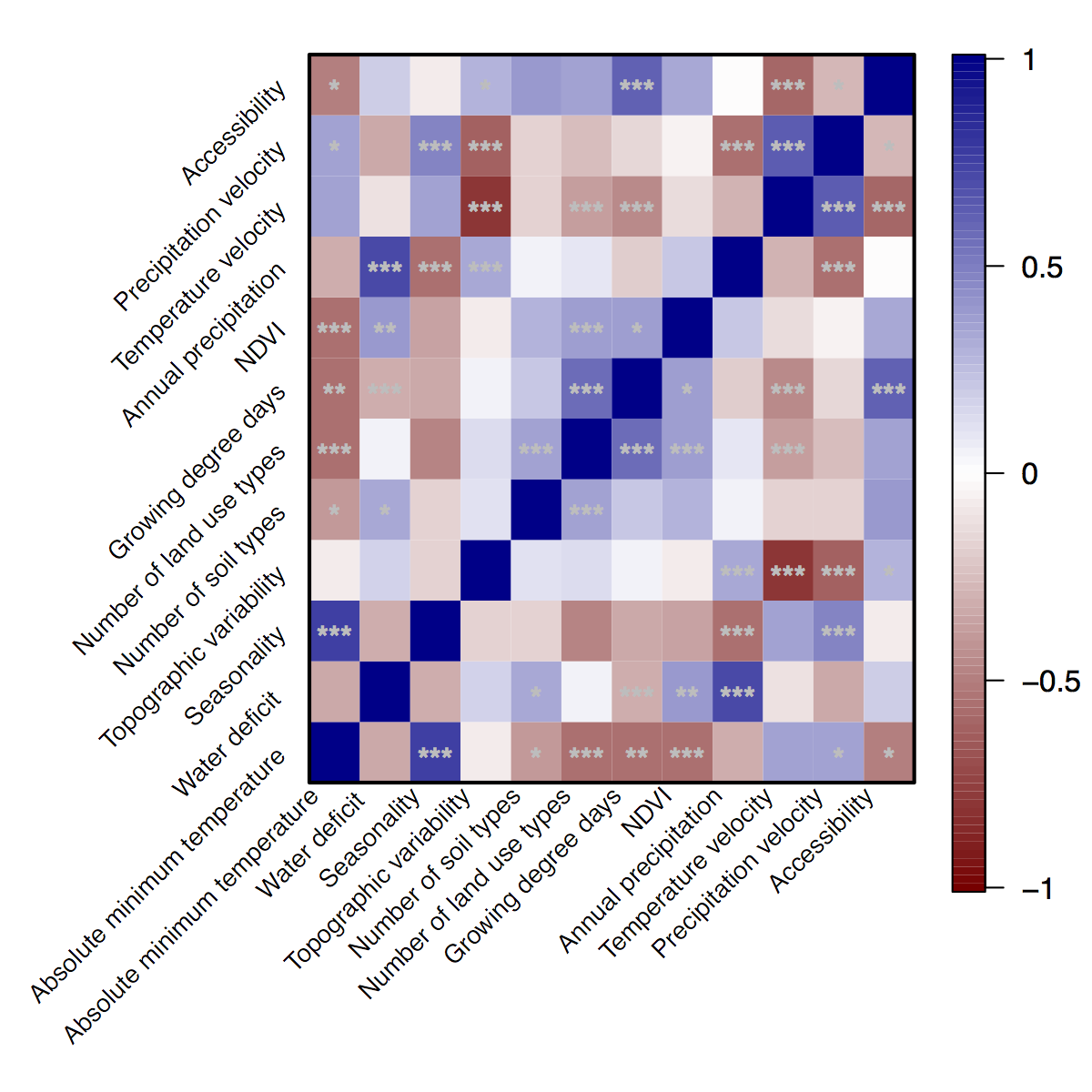 | | **Figure S8.** **Pearson correlated coefficients between environmental variables** used in this study. Significance of the correlation after accounting for spatial autocorrelation determined using [Dutilleul *et al.* (1993](#_ENREF_10)) correction. Significance levels: ***P < 0.001; **P < 0.01; *P < 0.05; blank is not significant. |  |

Spatially corrected pairwise correlations between variables indicated a weak correlation between variables (**Figure S8**) Most pairwise correlations were within the -0.25 to 0.25 range, with the highest correlation between topographic heterogeneity and temperature velocity (**Figure S8**).

**Appendix S4.** Individual macro-scale determinants of species richness standardized functional richness and dispersion.

**Geographic patterns of European trait diversity** – Richness standardized FD show similar geographical patterns for both richness standardized functional richness (F_Rich-STDZ_) and richness standardized functional dispersion (F_Disp-STDZ_). As indicated in **Figure S9**, maximum F_Rich-STDZ_ values are concentrated within the Mediterranean region most notably in the mountainous southern region (Pyrenees, Apennines, Alps, and Dinaric Alps). Maximum F_Disp-STDZ_ values also occur in the Iberian, Italian and Balkan peninsulas, most notably along the Apennines and the southern Carpathians (**Figure S9**). Furthermore, areas with the largest deviations from a null model (large positive or negative values) are areas in Southern Europe (highest positive deviations from a null model), and Scandinavian peninsulas (highest negative deviations from a null model). These patterns are more noticeable for F_Rich-STDZ_ than for F_Disp-STDZ_.

| 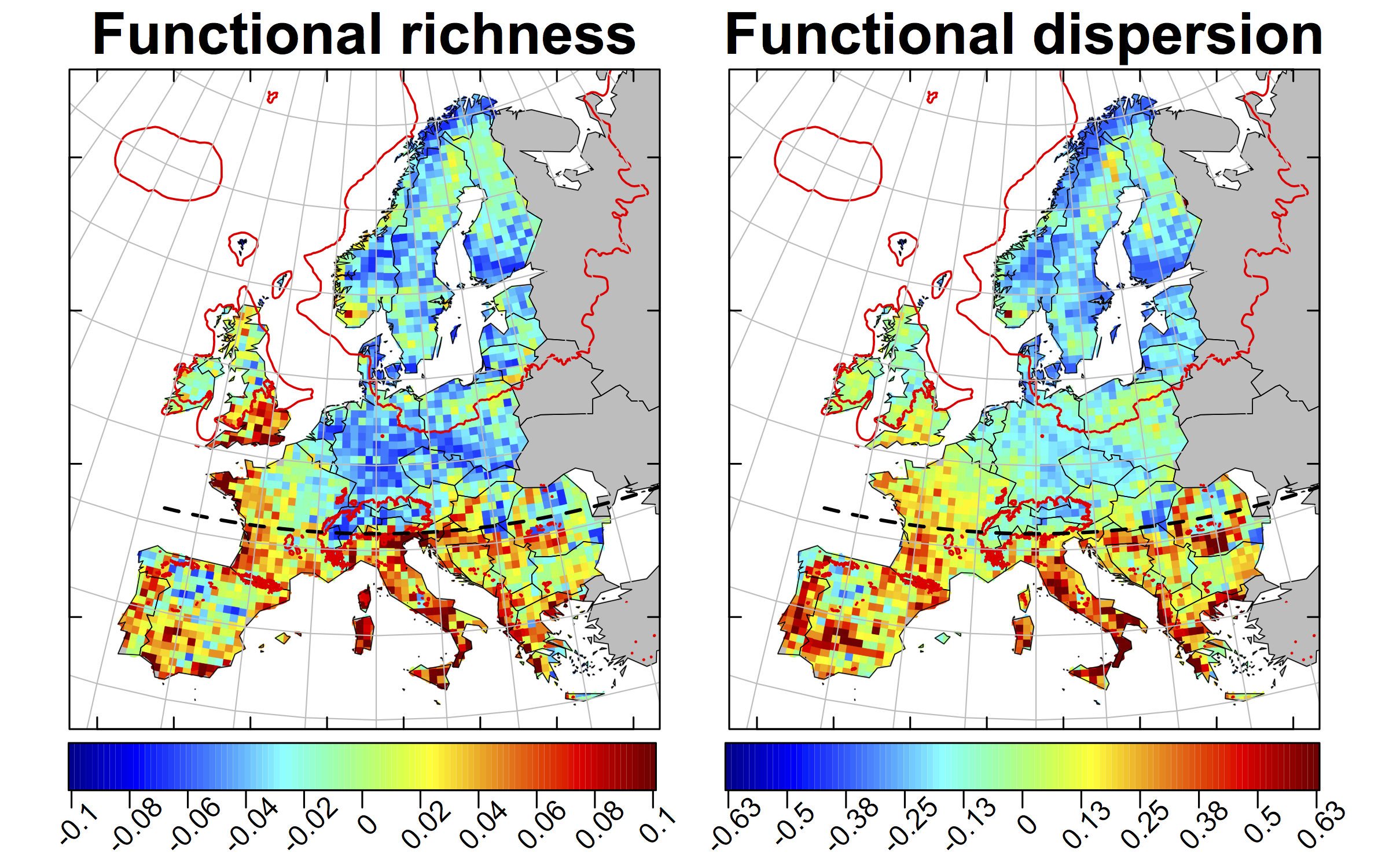 |
| --- |
| **Figure S9. The standardized functional diversity of European plants** for each of the Atlas Flora Europaeae grid cells (~50 km × 50 km). Evaluated species richness standardized functional diversity metrics represent the functional richness (left) and dispersion (right) of the trait space described by all evaluated traits. The dashed black line represents the 46° N latitude, which marks an estimate of the maximum northern limit of temperate tree full-glacial refugia. Areas delimited in red show the maximum ice area present over 21,000 years ago. |

**Individual macro-scale determinants of standardized functional richness and dispersion** – Comparison to the variance explained by historical variables (**Figure S10A, B**) shows the power of accessibility to LGM refugia as a predictor of both F_Rich-STDZ_ and F_Disp-STDZ_ in the region. The importance of accessibility to LGM refugia was confirmed by the multimodel support for this variable as a predictor of F_Rich-STDZ_ and F_Dis-STDZ_ across Europe (**Figure S10C, D**). As for non-standardized estimates of functional diversity, contemporary environmental variables showing the highest relative importance scores included growing degree-days, total precipitation, NDVI, water deficit and absolute minimum winter temperature for both F_Rich-STDZ_ and F_Disp -STDZ_ (**Figure S10C-D**). Furthermore, the significant variance explained, and high model support of accessibility, growing degree-days, and absolute minimum winter temperature was observed across the 10 imputed databases.

| 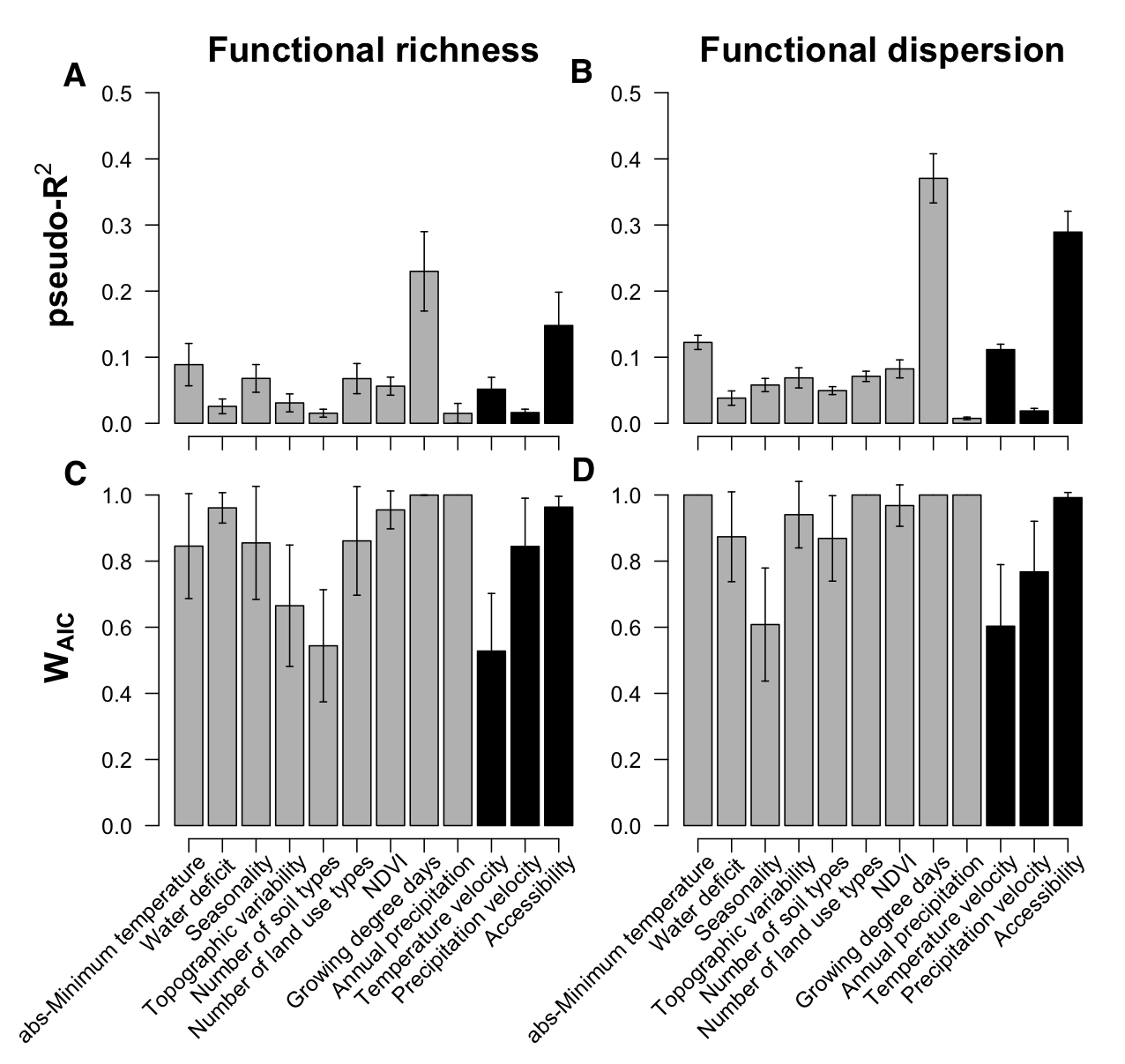 |
| --- |
| **Figure S10. Explained variance (A-B) and relative support (C-D) of historical (grey) and contemporary (black) environmental predictors associated with realized standardized functional richness and dispersion**. The bar height shows the mean explained variance and relative importance score across all ten estimated of standardized functional diversity, for each of the 12 evaluated predictors. Explained variance determined as Nagelkerke’s pseudo-*R*^2^ values ([Nagelkerke 1991](#_ENREF_25)) from single-predictor models with a unimodal response (lineal + quadratic terms). Relative support (*W_AIC_*) determined using [Burnham and Anderson (2002b](#_ENREF_5)) approach, where Akaike weights (*w_AIC_*) are summed across all models where the variable of interest was included as a linear or unimodal response. All relations between richness standardized functional diversity and environmental predictors determined using a spatial autoregressive error (SAR_error_) modeling approach (*See methods*). |

Standardized coefficients from the best SAR_error_ regressions explaining F_Rich-STDZ_ and F_Disp-STDZ_ (**Table S2**) provide strong support for the general importance of accessibility as the primary historical determinants of FD in the region. In the case of F_Rich-STDZ_, postglacial accessibility (positive effect) acts in conjunction with contemporary factors, specifically with growing degree-days (positive), annual precipitation (positive), water balance (negative), absolute minimum winter temperature (negative), and seasonality (positive). In the case of F_Disp-STDZ_ (**Table S2**), accessibility to Quaternary glacial-interglacial refugia (positive) was particularly influential in shaping observed patterns together with contemporary absolute minimum winter temperature (negative), growing degree-days (positive), annual precipitation (positive), and the number of land uses (negative).

| **Table S2. Model averaged standardized regression coefficients for contemporary and historical predictors as predictors of F_Rich_ and F_Disp_** for European plants in the Atlas Florae Europaeae. Coefficients were summarized using [Burnham and Anderson (2002a](#_ENREF_4)) model averaging approach, and indicate the *w_AIC_*-weighted mean of model averaged regression coefficients across all Imputed databases. Variables with significant support (mean-W_AIC_≥0.8) in bold. b: linear response. b^2^: quadratic response. Empty cells indicate that model average standardized coefficients were lower than 0.0005 | | | | | | |
| --- | --- | --- | --- | --- | --- | --- |
|  | **Functional**  **richness** | | **Functional**  **dispersion** | |  |  |
| **Contemporary predictors** | b | b^2^ | b | b^2^ |  |  |
| Absolute minimum winter temperature | **-0.177** | **0.253** | **-0.435** | **0.592** |  |  |
| Water balance | **-0.256** |  | -0.165 |  |  |  |
| Seasonality | **-0.793** | **0.780** | **-0.045** |  |  |  |
| Topographic heterogeneity | 0.039 |  | **0.106** |  |  |  |
| No of soil types | 0.015 |  | **0.051** |  |  |  |
| No of land uses | **-0.054** |  | **-0.172** |  |  |  |
| NDVI | **0.110** |  | **0.154** |  |  |  |
| Growing degree days | **0.401** |  | **0.548** |  |  |  |
| Annual precipitation | **0.345** |  | **0.252** |  |  |  |
| **Historical predictors** |  |  |  |  |  |  |
| Temperature velocity | 0.033 |  | -0.030 |  |  |  |
| Precipitation velocity | -0.033 |  | 0.054 |  |  |  |
| Accessibility | **0.040** | 0.142 | **0.243** |  |  |  |

***Appendix S5*–**Association between univariate (trait range and dispersion) and multivariate metrics of functional diversity.

**Geographic patterns of European trait diversity–** Regions exhibiting high trait range and dispersion were located primarily in areas considered to have experienced smaller glacial-interglacial climatic changes and south of the northern limit our estimates of temperate species refugia (approximately 46°N). As is also the case for F_Rich_, trait range values are concentrated within the Mediterranean region (**Figure S11**), most notably central Europe and the mountainous southern region (Pyrenees, Apennines, Alps, and Dinaric Alps). Similarly, areas with maximum trait deviation show similar patterns as those observed for F_Disp_ on the west coast of Scandinavia and along the Iberian, Italian and Balkan peninsulas, most notably along the Apennines and southern Carpathians showing the widest dispersion (**Figure S11**).

| 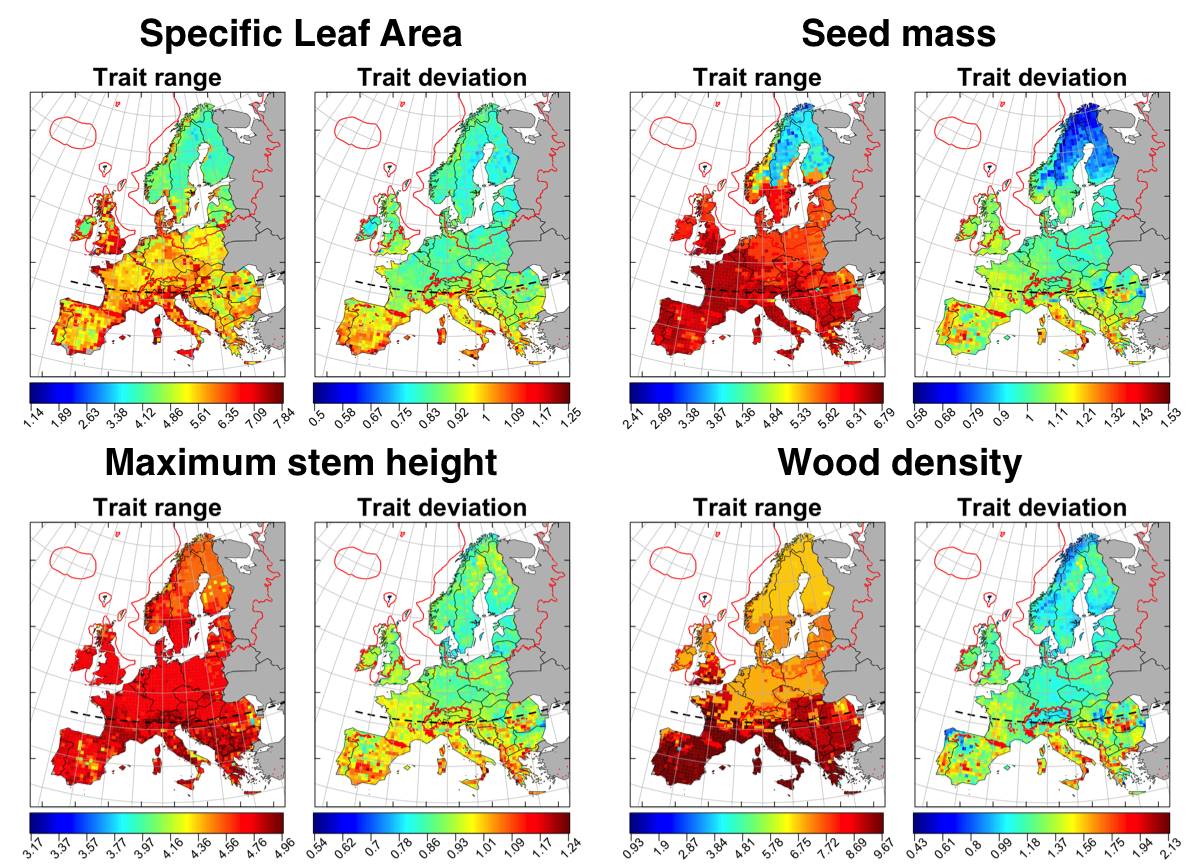 |
| --- |
| **Figure S11.** **Trait range and deviation across Europe** for the four continuous traits in the analyses: specific leaf area, maximum stem height, seed mass and wood density. Dashed black line represents the 46° N latitude, an estimate of the maximum northern limit of temperate tree full-glacial refugia. Red areas show the maximum ice over 21.000yrs-ago. |

Trait range relative importance as a determinant of F_Rich_, and of trait deviation as a determinant of F_Disp_ showed the importance of all three evaluated attributes. Evidence for this is the high support of these variables as shown by summed Akaike weights (*w*_AIC_; **Table S4**) higher than 80% of the range and deviation of evaluated traits. For F_Rich_, *w*_AIC_ of SLA, and SWT ranges were 100% in all the evaluate imputations. For F_Disp_, *w*_AIC_ of SWT and WD dispersion was also 100% in all the evaluate imputations. Evaluation of the mean accords all imputed datasets of model averaged standardized regression coefficients (**Table S4**) shows that F_Rich_ is positively associated with all trait ranges, with SLA and SWT ranges having the strongest impact. In the F_Disp_ case, there is a positive association with the dispersion of all traits, but variation in SWT and WD had the most substantial effects. The association of F_Rich_ with SWT and SLA range indicate that the trait space is maximized in areas with a broad array of dispersal strategies and alternative carbon capturing strategies. Meanwhile, the positive relation with SWT and WD show that functional space filling is maximized in areas with a diverse assortment of growth forms (woody and non-woody) and dispersal strategies.

| **Table S4. Model averaged standardized regression coefficients for trait range and deviation as explanatory variables of F_Rich_ and F_Disp_**. Coefficients were summarized using [Burnham and Anderson (2002a](#_ENREF_4)) model averaging approach, and indicate the *w_AIC_*-weighted mean of model averaged regression coefficients across all Imputed databases. | | | | | | |
| --- | --- | --- | --- | --- | --- | --- |
|  | **Functional**  **richness** | | **Functional**  **dispersion** | |  |  |
|  | b | W_AIC_ | b | W_AIC_ |  |  |
| Specific leaf area  (cm^2^*g^-1^) | 0.368 | 100% | 0.133 | 99% |  |  |
| Maximum stem height  (m) | 0.022 | 84% | 0.108 | 85% |  |  |
| Seed mass  (mg) | 0.484 | 100% | 0.385 | 100% |  |  |
| Wood/Stem density  (kg*m^-3^) | 0.126 | 86% | 0.467 | 100% |  |  |

**References**

1.

Baker, T.R., Phillips, O.L., Laurance, W.F., Pitman, N.C.A., Almeida, S., Arroyo, L. *et al.* (2009). Do species traits determine patterns of wood production in Amazonian forests? *Biogeosciences*, 6, 297-307.

2.

Baker, T.R., Phillips, O.L., Malhi, Y., Almeida, S., Arroyo, L., Di Fiore, A. *et al.* (2004). Variation in wood density determines spatial patterns in Amazonian forest biomass. In: *Global Change Biol*, pp. 545-562.

3.

Bruggeman, J., Heringa, J. & Brandt, B.W. (2009). PhyloPars: estimation of missing parameter values using phylogeny. *Nucleic Acids Res*, 37, W179-W184.

4.

Burnham, K.P. & Anderson, D.R. (2002a). *Model selection and multi-model inference: a practical information-theoretic approach*. Springer.

5.

Burnham, K.P. & Anderson, D.R. (2002b). *Model selection and multimodel inference: a practical information-theoretic approach*. Springer.

6.

Büttner, G., Feranec, J., Jaffrain, G., Mari, L., Maucha, G. & Soukup, T. (2004). The CORINE land cover 2000 project. In: *EARSeL eProceedings* (ed. Reuter, R). EARSeL Paris, pp. 331–346.

7.

Buuren, S. & Groothuis-Oudshoorn, K. (2011). MICE: Multivariate imputation by chained equations in R. *J Stat Softw*, 45, 1-67.

8.

Chave, J., Coomes, D., Jansen, S., Lewis, S.L., Swenson, N.G. & Zanne, A.E. (2009). Towards a worldwide wood economics spectrum. *Ecology Letters*, 12, 351-366.

9.

Diniz, J.A.F., Bini, L.M., Sakamoto, M. & Brusatte, S.L. (2014). Phylogenetic Eigenvector Regression in Paleobiology. *Rev Bras Paleontolog*, 17, 105-122.

10.

Dutilleul, P., Clifford, P., Richardson, S. & Hemon, D. (1993). Modifying the t test for assessing the correlation between two spatial processes. *Biometrics*, 49, 305-314.

11.

Falster, D.S. & Westoby, M. (2003). Plant height and evolutionary games. *Trends in Ecology & Evolution*, 18, 337-343.

12.

FAO (2014). Food Agriculture Organization of the United Nations GEONETWORK. FAO Rome, Italy.

13.

Forsythe, W.C., Rykiel Jr, E.J., Stahl, R.S., Wu, H.-i. & Schoolfield, R.M. (1995). A model comparison for daylength as a function of latitude and day of year. *Ecological Modelling*, 80, 87-95.

14.

Heitjan, D.F. & Little, R.J. (1991). Multiple imputation for the fatal accident reporting system. *Applied Statistics*, 13-29.

15.

Hijmans, R., Cameron, S., Parra, J., Jones, P. & Jarvis, A. (2005). Very high resolution interpolated climate surfaces for global land areas. *International Journal of Climatology*, 25, 1965-1978.

16.

Horton, N.J. & Lipsitz, S.R. (2001). Multiple imputation in practice: comparison of software packages for regression models with missing variables. *The American Statistician*, 55, 244-254.

17.

Jones, A., Montanarella, L. & Jones, R. (2005). *Soil atlas of Europe*. European Commission.

18.

Kattge, J., Díaz, S., Lavorel, S., Prentice, I.C., Leadley, P., BÖNisch, G. *et al.* (2011). TRY – a global database of plant traits. *Global Change Biology*, 17, 2905-2935.

19.

Kleyer, M., Bekker, R.M., Knevel, I.C., Bakker, J.P., Thompson, K., Sonnenschein, M. *et al.* (2008). The LEDA Traitbase: a database of life-history traits of the Northwest European flora. *Journal of Ecology*, 96, 1266-1274.

20.

Leishman, M.R., Wright, I., Moles, A.T. & Westoby, M. (2000). The evolutionary ecology of seed size. In: *Seeds: The Ecology of Regeneration in Plant Communities* (ed. Fenner, M). CABI Publishing Wallingford, UK,, pp. 31–57.

21.

Leroy, S.A.G. & Arpe, K. (2007). Glacial refugia for summer-green trees in Europe and south-west Asia as proposed by ECHAM3 time-slice atmospheric model simulations. *Journal of Biogeography*, 34, 2115-2128.

22.

Liu, K., Eastwood, R.J., Flynn, S., Turner, R.M. & Stuppy, W.H. (2008). Seed Information Database (release 7.1, May 2008) - <http://www.kew.org/data/sid>.

23.

Malhi, Y., Wood, D., Baker, T.R., Wright, J., Phillips, O.L., Cochrane, T. *et al.* (2006). The regional variation of aboveground live biomass in old-growth Amazonian forests. *Global Change Biology*, 12, 1107-1138.

24.

Moles, A.T. & Westoby, M. (2006). Seed size and plant strategy across the whole life cycle. *Oikos*, 113, 91-105.

25.

Nagelkerke, N.J.D. (1991). A Note on a General Definition of the Coefficient of Determination. *Biometrika*, 78, 691-692.

26.

Ohlemüller, R., Gritti, E.S., Sykes, M.T. & Thomas, C.D. (2006). Towards European climate risk surfaces: the extent and distribution of analogous and non-analogous climates 1931–2100. *Global Ecology and Biogeography*, 15, 395-405.

27.

Ordonez, A. & Olff, H. (2013). Do alien plant species profit more from high resource supply than natives? A trait-based analysis. *Global Ecology and Biogeography*.

28.

Ordonez, A., Wright, I.J. & Olff, H. (2010). Functional differences between native and alien species: a global-scale comparison. *Functional Ecology*, 24, 1353-1361.

29.

Rubin, D.B. (2009). *Multiple imputation for nonresponse in surveys*. Wiley. com.

30.

Sandel, B., Arge, L., Dalsgaard, B., Davies, R.G., Gaston, K.J., Sutherland, W.J. *et al.* (2011). The influence of late Quaternary climate-change velocity on species endemism. *Science*, 334, 660-664.

31.

Schafer, J.L. (1999). Multiple imputation: a primer. *Statistical methods in medical research*, 8, 3-15.

32.

Schenker, N. & Taylor, J.M. (1996). Partially parametric techniques for multiple imputation. *Computational Statistics & Data Analysis*, 22, 425-446.

33.

Shan, H., Kattge, J., Reich, P., Banerjee, A., Schrodt, F. & Reichstein, M. (2012). Gap Filling in the Plant Kingdom---Trait Prediction Using Hierarchical Probabilistic Matrix Factorization. *Proceedings of the 29th International Confer- ence on Machine Learning, Edinburgh, Scotland, UK, 2012.*

34.

Swenson, N. & Enquist, B. (2007). Ecological and evolutionary determinants of a key plant functional trait: Wood density and its community-wide variation across latitude and elevation. In: *American Journal of Botany*, pp. 451-459.

35.

Swenson, N.G. (2014). Phylogenetic imputation of plant functional trait databases. *Ecography*, 37, 105-110.

36.

Thornthwaite, C.W. (1948). An approach toward a rational classification of climate. *Geographical Review*, 38, 55-94.

37.

USDA-NRCS (2013). The PLANTS Database (<http://plants.usda.gov>, 27 August 2013). (ed. Team, NPD) Greensboro, NC 27401-4901 USA.

38.

Westoby, M., Falster, D.S., Moles, A.T., Vesk, P.A. & Wright, I.J. (2002). Plant ecological strategies: Some leading dimensions of variation between species. *Annual Review of Ecology and Systematics*, 33, 125-159.

39.

Wright, I.J., Reich, P.B., Westoby, M., Ackerly, D.D., Baruch, Z., Bongers, F. *et al.* (2004). The worldwide leaf economics spectrum. *Nature*, 428, 821-827.
